# Highly Cited Research on Botulinum Toxin: A Bibliometric Analysis of Clinical Trends, Influential Evidence, and Healthcare-Relevant Applications (1991–2022)

**DOI:** 10.3390/healthcare14111480

**Published:** 2026-05-27

**Authors:** Yuh-Shan Ho, Lucas Alexandre de Sousa Mendes, Giancarlo De la Torre Canales, Nikolaos Christidis

**Affiliations:** 1 CT HO Trend, 3F.-7, No. 1, Fuxing N. Rd., Songshan Dist., Taipei 105611, Taiwan; dr_ysho@hotmail.com; 2 Department of Dentistry, Ingá University Center, Uningá, Maringá 87035-510, PR, Brazil; drlucassousamendes.cd@outlook.com; 3 Division of Oral Rehabilitation, Department of Dental Medicine, Karolinska Institutet, SE-14104 Huddinge, Sweden; giancarlo.de.la.torre.canales@ki.se

**Keywords:** botulinum toxin, bibliometric analysis, highly cited publications, clinical applications, spasticity, dystonia, chronic pain, healthcare research

## Abstract

**Highlights:**

**What are the main findings?**

- Highly cited botulinum toxin research clusters around five major clinical areas: spasticity, dystonia, chronic pain, urology, and aesthetic medicine.
- High-impact publications are predominantly produced through international and multi-author collaborations, with strong contributions from industry-linked research.

**What are the implications of the main findings?**

- The evolution of highly cited research may reflect expanding clinical indications and growing integration of botulinum toxin across multiple healthcare disciplines.
- Understanding influential research patterns may support evidence-based decision-making, guide future research priorities, and inform clinical and healthcare policy development.

**Abstract:**

**Background/objectives:** Botulinum toxin is widely used across medical specialties, with expanding indications in neurology, pain management, urology, and aesthetic medicine. Highly cited publications can reflect influential evidence and clinical priorities within healthcare. This study aimed to identify and characterize highly cited research on botulinum toxin as indicators of influential evidence within the field, with a focus on clinical trends, collaboration patterns, and healthcare-relevant applications. **Methods:** A bibliometric analysis was conducted using the Science Citation Index Expanded (SCI-EXPANDED) within the Web of Science Core Collection. Publications from 1991 to 2022 with ≥100 citations (*TC*_2024_ ≥ 100) were included. Data on publication characteristics, authorship, institutional and country contributions, and citation impact were extracted. Bibliometric indicators, including total publications, citations per publication, and the *Y*-index, were applied. Keyword and title analyses were used to identify major research themes. **Results:** A total of 643 highly cited articles were identified, with 118,175 citations. The United States was the leading contributor, and industry-linked research, particularly from Allergan, demonstrated high citation impact. Five major clinical themes emerged: spasticity management, dystonia and movement disorders, chronic pain, urinary disorders, and cosmetic applications. Highly cited research was predominantly produced through collaborative teams, most commonly involving two to six authors, and international collaborations were associated with higher citation impact. **Conclusions:** Highly cited botulinum toxin research may partially reflect evolving clinical demand and interdisciplinary use across healthcare fields. The prominence of collaborative and industry-linked research highlights the role of translational and practice-driven innovation. These findings provide insight into influential evidence and may support future research prioritization, interdisciplinary collaboration, and evidence-based decision-making in clinical and healthcare contexts.

## 1. Introduction

Botulinum toxin, known as one of the most potent biological toxins identified, is a neurotoxin produced from the fermentation of the anaerobic, Gram-positive bacterium *Clostridium botulinum* [1]. Among the different serotypes, type A is the most widely used in clinical practice due to its potency and therapeutic profile. Botulinum toxins act at four different sites in the body: the neuromuscular junction, autonomic ganglia, postganglionic parasympathetic nerve endings, and postganglionic sympathetic nerve endings that release acetylcholine [1,2]. Scott was the first to show that botulinum toxin type A can effectively manage strabismus in humans [3]. Following this, the toxin gained approval for treating various spasticity-related disorders and several other medical issues [2]. Today, its use spans nearly every medical sub-specialty. This broad clinical adoption highlights the importance of understanding how evidence has evolved to inform clinical practice, healthcare decision-making, and resource allocation across different medical disciplines. In 2002, the U.S. Food and Drug Administration (FDA) granted approval for Botox^®^ (Botulinum toxin-A) to be used cosmetically to temporarily diminish frown lines on the forehead and between the eyebrows and in 2010 as a preventive treatment for chronic migraine [4,5]. Therefore, botulinum toxin A rapidly expanded in clinical use because of its efficacy, tolerability, and minimally invasive nature [6].

The idea of “highly cited” publications in the field of mathematics was first introduced in the early 1970s by Eugene Garfield [7]. In his subsequent work, Garfield observed that articles receiving 100 or more citations were predominantly found in clinical or general medical journals [8]. Later, he emphasized that many foundational studies within specific disciplines tend to maintain high citation levels over long periods [9]. In later years, highly cited publications became recognized as indicators of excellence and international visibility in research output [10,11]. In bibliometric research, highly cited publications are considered indicators of sustained scientific influence and knowledge diffusion within a field [10,11]. In healthcare contexts, they may also reflect evidence that contributes to clinical guidelines, therapeutic strategies, and translational development. However, previous bibliometric analyses have primarily mapped the overall publication landscape of botulinum toxin research, focusing on publication volume, geographic distribution, and general trends rather than identifying the most influential studies shaping clinical and translational practice [12], without specifically examining the subset of highly cited publications that may represent the most influential evidence. This limits understanding of how scientific knowledge translates into clinical practice and healthcare relevance. Focusing on highly cited publications therefore enables identification of influential research patterns, dominant clinical themes, and key contributors that have shaped the field.

However, citation counts in the Web of Science Core Collection (WoSCC) are dynamic and continually updated, which can complicate reproducibility in bibliometric analyses. To address this, the present study used *TC*_2024_, defined as the total number of citations received from the year of publication through the end of 2024. *TC*_2024_ provides a fixed, time-bound citation count, enhancing the consistency and replicability of bibliometric studies, unlike fluctuating real-time citation metrics [13].

Using this framework, publications with a *TC*_year_ of 100 or more citations are categorized as “highly cited” [14]. This standardized definition has since gained broad acceptance across various research fields.

In this study, publications were identified as “highly cited” if they received 100 or more citations in the WoSCC from their year of publication through the end of 2024. Unlike the previous bibliometric mapping that examined the entire publication landscape [12], this study isolates the top-cited segment of the literature. The rationale for this approach is that highly cited papers often act as intellectual milestones, reflecting both scientific excellence and translational relevance. This methodological narrowing enables a qualitative interpretation of impact patterns rather than a purely quantitative trend analysis with particular emphasis on identifying clinically relevant research patterns and their implications for healthcare practice and interdisciplinary care. Accordingly, the aim of this study was to identify and characterize highly cited botulinum toxin publications, with a focus on clinical trends, collaboration patterns, and healthcare-relevant applications, and to identify the subset of studies that have most strongly influenced clinical adoption and interdisciplinary use of botulinum toxin. The remainder of this article is structured as follows. Section 2 describes the data source, search strategy, and bibliometric indicators. Section 3 presents the results. Section 4 discusses the findings and their implications, and Section 5 provides the conclusions.

## 2. Materials and Methods

### 2.1. Data Source and Search Strategy

Building upon our previous global bibliometric assessment of botulinum toxin research [12], this study employed a refined dataset drawn from the Web of Science Core Collection (SCI-EXPANDED), limited exclusively to publications that had achieved at least 100 citations based on *TC*_2024_ (total citations from publication to the end of 2024). This study was conducted as an original bibliometric analysis of the published literature rather than a narrative or systematic review. To enhance transparency, reproducibility, and methodological rigor, the study was designed in accordance with the BIBLIO checklist for bibliometric studies in biomedical research (Table S1) [15].

Data were retrieved from the Clarivate Analytics Web of Science Core Collection (WoSCC), the online version of the Science Citation Index Expanded (SCI-EXPANDED) (data updated on 1 May 2025). All data were retrieved on the same date to ensure consistency of citation counts. To optimize the capture of both clinical and experimental studies, a topic (TS) search was performed using Boolean operators and phrase searching, covering publications from 1991 to 2024. The topic (TS) field includes title, abstract, author keywords, and *Keywords Plus*.

The final search string used in the Web of Science Core Collection was as follows: TS = (“botulinum toxin” OR “botulinum neurotoxin” OR “onabotulinumtoxinA” OR “BOTOX” OR “BoNT-A” OR “BTX-A” OR “botulinum toxins” OR “botulinum A” OR “DYSPORT” OR “incobotulinumtoxinA” OR “abobotulinumtoxinA” OR “onabotulinum toxin” OR “clostridium botulinum type” OR “botulinum type” OR “XEOMIN” OR “botulinum B” OR “BTX-B” OR “botulinum C” OR “botulinum D” OR “botulinum E” OR “botulinum F” OR “botulinum G” OR “botulinum neurotoxin type A” OR “DaxibotulinumtoxinA” OR “toxin botulinum” OR “Vistabel” OR “Nabota”).

To ensure comprehensive coverage of both clinical and experimental research, no restrictions were applied regarding study design. The search strategy was not structured using the PICOC framework, as the study aimed to capture a broad bibliometric landscape rather than a narrowly defined clinical question. The search terms were combined using Boolean operators as described in the final search string above, designed to capture variations in terminology and nomenclature used across the literature.

### 2.2. Inclusion Criteria and Definition of Highly Cited Articles

**Figure 1:**
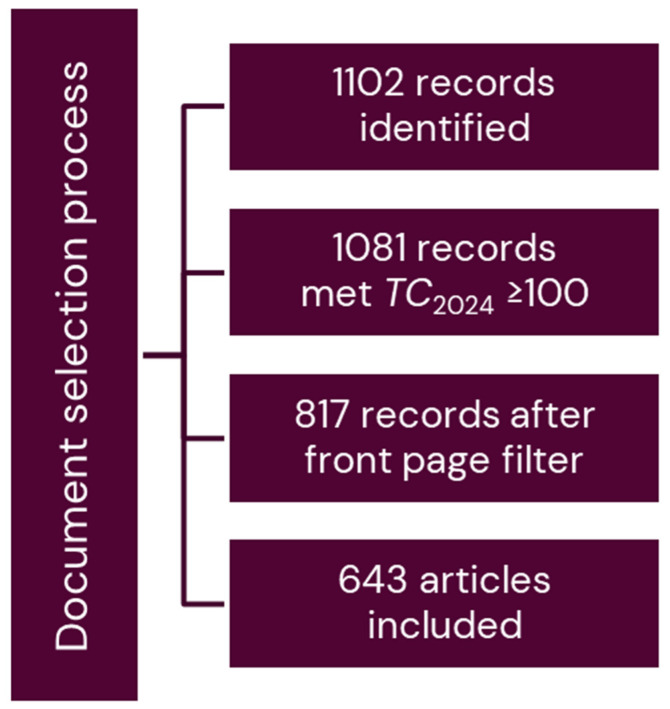
Flowchart of document selection process.

A total of 1102 documents containing the defined search keywords and achieving at least 100 citations in WoSCC were retrieved. In this study, publications with *TC*_2024_ ≥ 100 were defined as “highly cited”. The document selection process is shown in Figure 1.

Following data verification, 1081 documents (98% of the initial dataset) met this criterion. For subsequent analysis, only articles were included, as they typically provide comprehensive scientific content [16].

To refine the search strategy the “front page” filter (title, abstract, and author keywords) was applied to improve relevance by ensuring that botulinum toxin was a central focus of the study rather than a peripheral mention [13]. This resulted in 817 highly cited botulinum toxin-relevant documents, representing 76% of the 1081 documents. A manual screening step was not performed; however, the application of the “front page” filter has been previously proposed as an effective method for improving relevance in bibliometric studies [13]. However, the use of topic search (TS), which includes Keywords Plus, may capture marginally related publications. The application of the “front page” filter was intended to mitigate this limitation by prioritizing studies where botulinum toxin was central to the content.

### 2.3. Data Extraction and Variables

Complete records, including annual citation counts, were downloaded into Microsoft 365 Excel for further analysis. Data extraction included publication year, document type, language, journal, author names, institutional affiliations, country of origin, and author keywords. Journal impact factors (*IF*_2023_) were obtained from the 2024 edition of the Journal Citation Reports.

To ensure data consistency, affiliation standardization was performed. For example, regional entities such as England, Scotland, and Northern Ireland were grouped under the United Kingdom (UK), and institutions with multiple campuses (e.g., Mayo Clinic in Rochester, Phoenix, Scottsdale, and Jacksonville) were consolidated into the single entity “Mayo Clinic” [17].

In WoSCC, the reprint author is listed as the corresponding author; therefore, the term “corresponding author” was used consistently [17]. First and corresponding authors were considered key contributors, reflecting primary intellectual input [18]. In single-author publications, the author was assigned both roles [17]. In cases involving multiple corresponding authors, all authors and affiliations were recorded [17].

### 2.4. Bibliometric Indicators and Analysis

The evaluation of publications was conducted using established bibliometric indicators. These included:

- Total publications (*TPs*);
- Independent publications (*IPs*) and collaborative publications (*CPs*);
- First-author publications (*FPs*);
- Corresponding-author publications (*RPs*);
- Single-author publications (*SPs*).

Citation indicators included:

- *C*_year_: the number of citations received in a specific year (e.g., *C*_2024_) [19];
- *TC*_2024_: the number of total citations from publication year to 2024 [20];
- *CPP*_2024_: the number of citations per publication [21].

These indicators provide stable and reproducible measures, in contrast to dynamic real-time citation counts [22].

The *Y*-index, introduced by Ho [19], was applied to evaluate author contributions based on first- and corresponding-author publications. This indicator consists of two parameters—*j* (publication potential) and *h* (authorship characteristics)—allowing a more nuanced assessment of research performance [14,19].

Additionally, six publication indicators and corresponding citation indicators were applied to assess country- and institution-level performance [23].

### 2.5. Thematic and Keyword Analysis

Finally, word frequency analysis of article titles and author keywords was conducted to identify major research themes. This method has been proposed as an effective approach for mapping research topics and thematic trends within the scientific literature [24].

### 2.6. Generative Artificial Intelligence in Manuscript Preparation

Generative artificial intelligence (GenAI) tools were not used in the design, analysis, or writing of this manuscript.

## 3. Results

### 3.1. Document Type and Publication Characteristics

A total of 817 highly cited botulinum toxin documents in the SCI-EXPANDED database, each with *TC*_2024_ values of 100 or more citations, were identified. Table 1 presents the characteristics of the eight identified document types, including the total number of publications (*TP*), *APP*, and *CPP*_2024_. Among these, articles were the most prevalent document type, comprising 643 records (79% of the 817 highly cited documents), with an *APP* of 6.2 average number of authors per publication. Because Web of Science document types may overlap, the summed total exceeds the number of unique publications. Highly cited reviews, while fewer in number, demonstrated the highest impact, with a *CPP*_2024_ of 203 citations per publication, slightly exceeding that of articles, which had a *CPP*_2024_ of 184 citations. This finding indicates that highly cited reviews achieved citation impact comparable to that of highly cited articles in botulinum toxin research. A total of 162 highly cited reviews were published in a wide range of 108 journals. The majority appeared in *Movement Disorders* (*IF*_2023_ = 7.4) (10 reviews; 6.2% of 162 highly cited reviews), with a *CPP*_2024_ of 173 citations per publication, followed by *Cochrane Database of Systematic Reviews* (*IF*_2023_ = 8.8) (nine reviews; 5.6% of 162 highly cited reviews), with a *CPP*_2024_ of 109 citations. Two of the four classic publications with *TC*_2024_ of 1000 or more citations [25] in botulinum toxin research were reviews: “Chapter 4: European guidelines for the management of chronic nonspecific low back pain” by Airaksinen et al. (2006), with a *TC*_2024_ of 1723 citations [26], and “Botulinum toxin as a biological weapon: Medical and public health management” by Arnon et al. (2001), with a *TC*_2024_ of 1008 citations [27]. The most frequently cited note was “Identification of the nerve terminal targets of botulinum neurotoxin serotypes A, D, and E” by Schiavo et al. (1993), with a *TC*_2024_ of 375 citations [28].

Finally, 642 highly cited articles related to botulinum toxin research were published in English and one in Spanish, and these 643 articles were selected for further detailed analysis.

### 3.2. Publication Distribution

**Figure 2:**
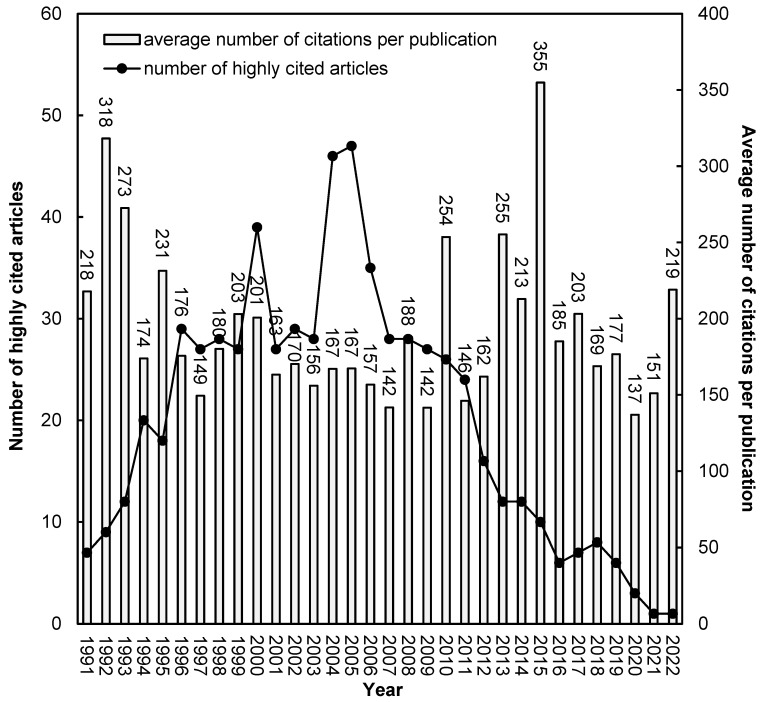
Annual number of highly cited articles and corresponding *CPP*_2024_ values.

In total, 643 highly cited botulinum toxin-related articles published between 1991 and 2022 were identified, with a combined *TC*_2024_ of 118,175 citations, averaging 184 citations per article. The highest *TC*_2024_ observed was 2042 citations for a single article. Figure 2 illustrates the annual distribution of these 643 articles along with their corresponding *CPP*_2024_ values.

The year 2015, with 10 articles, recorded the highest *CPP*_2024_, with 355 citations per publication. This peak was attributable to the classic botulinum toxin-related article titled “Pharmacotherapy for neuropathic pain in adults: A systematic review and meta-analysis” by Finnerup et al. (2015), which received a *TC*_2024_ of 2042 citations and ranked as the 1st most cited article in the dataset [29].

The year 2005 emerged as the most productive in terms of generating highly cited articles in this domain. From 1991 to 2024, the earliest seven highly cited articles were published in 1991, with five of them explicitly including a botulinum toxin-related keyword in their titles. The most recent highly cited publication identified was “Migraine” by Ferrari et al. (2022), which achieved a *TC*_2024_ of 219 citations, ranking 141st in the dataset [30].

### 3.3. Web of Science Categories and Journals in SCI-EXPANDED

A total of 217 journals in 59 Web of Science categories in SCI-EXPANDED published highly cited articles related to botulinum toxin. Table 2 presented the top 10 most productive Web of Science categories. The category of clinical neurology was the predominant category, housing 211 journals in 2023 and accounting for 29% of the total articles (187 out of 643 articles). Compared with the top ten categories in Table 2, 34 highly cited articles published in the category of multidisciplinary sciences had the highest *CPP*_2024_ of 260 citations per publication while 84 articles in surgery had a *CPP*_2024_ of 142 citations. Articles published in category of gastroenterology and hepatology had the highest *APP* of 10 authors per publication while dermatology had an *APP* of 3.8 authors.

Table 3 presents the top 10 most productive journals, each publishing 15 or more highly cited articles. Among these, four journals belong to the category of clinical neurology. *Neurology* (*IF*_2023_ = 7.7) published the highest number of highly cited articles, with 37 articles, accounting for 5.8% of the total 643 highly cited articles. Articles published in *Headache* (*IF*_2023_ = 5.4) achieved the highest *CPP*_2024_, with 240 citations per publication, while the 18 highly cited articles in *Plastic and Reconstructive Surgery* (*IF*_2023_ = 3.2) had a *CPP*_2024_ of 143 citations per publication. The average number of authors per publication (*APP*) ranged from 7.7 authors per publication in *European Urology* to 3.8 authors in *Plastic and Reconstructive Surgery*.

Based on the 2023 Journal Impact Factors (*IF*_2023_), the top eight journals publishing highly cited articles related to botulinum toxin were identified. *The Lancet* ranked first with the highest *IF*_2023_ of 98.4, contributing two highly cited articles. It was followed by *The New England Journal of Medicine* (*IF*_2023_ = 96.2), with seven articles, and *The BMJ–British Medical Journal* (*IF*_2023_ = 93.6), with one article. *Nature Reviews Disease Primers* (*IF*_2023_ = 76.9) published two articles, while *JAMA–Journal of the American Medical Association* (*IF*_2023_ = 63.1) contributed three articles. These five journals occupy the top five positions among 167 journals in the general and internal medicine category. Additionally, *Nature Medicine* (*IF*_2023_ = 58.7) published two highly cited articles and ranked first among 135 journals in the category of research and experimental medicine and among 284 journals in biochemistry and molecular biology. *Nature* (*IF*_2023_ = 50.4) contributed nine articles and was the leading journal among 72 titles in the multidisciplinary sciences category. Finally, *The Lancet Neurology* (*IF*_2023_ = 46.5) published four articles and was ranked first among 257 journals in the clinical neurology category.

### 3.4. Countries, Institutions, and Authors

Within the SCI-EXPANDED database, two out of 643 highly cited botulinum toxin-related articles (0.31%) lacked author affiliation information. The remaining 641 articles were authored by researchers affiliated with institutions in 45 different countries. Of these, 462 articles (72%) were classified as single-country articles originating from 26 countries, with a *CPP*_2024_ of 168 citations per publication. The remaining 179 articles (28%) were internationally collaborative, involving co-authors from 44 countries, and showed an obviously higher citation impact, with a *CPP*_2024_ of 224 citations.

Table 4 summarizes data for the 10 most productive countries, each with at least 13 highly cited articles. The list includes six European countries, two from the Americas, and one each from Asia and Oceania, respectively. South Africa was the one country in Africa with one internationally collaborative highly cited article. Among the top countries, five—France, Australia, Switzerland, Japan, and Belgium—had no single-author highly cited articles. The USA dominated all six publication indicators:

1. Total publications (*TPs*): A total of 347 highly cited articles (54% of 641 highly cited articles);
2. Independent publications (*IP*_Cs_): A total of 239 articles (52% of 462 single-country highly cited articles);
3. Collaborative publications (*CP*_Cs_): A total of 108 articles (60% of 179 internationally collaborative highly cited articles);
4. First-author publications (*FPs*): A total of 300 articles (47% of 641 first-author highly cited articles);
5. Corresponding-author publications (*RPs*): A total of 299 articles (48% of 629 corresponding-author highly cited articles);
6. Single-author publications (*SPs*): A total of 17 highly cited articles (53% of 32 single-author highly cited articles).

Among the top 10 countries, France, with a *TP* of 43 articles and a *CP*_C_ of 27 articles, had the highest *CPP*_2024_ of 278 and 365 citations per publications for total articles and internationally collaborative articles, respectively. Switzerland, with an *IP*_C_ of three articles, an *FP* of 18 articles, and an *RP* of 16 articles, had the highest *CPP*_2024_ of 194, 259, and 272 citations per publication for single-country articles, first-author articles, and corresponding-author articles, respectively. Canada, with an *SP* of one article, had the highest *CPP*_2024_ of 555 citations per publication for single-author articles.

Of the 641 botulinum toxin-related highly cited articles with institutional affiliation information in the SCI-EXPANDED database, 210 articles (33%) were published by a single institution and had a *CPP*_2024_ of 167 citations per publication. The remaining 431 articles (67%) were the result of inter-institutional collaborations, which recorded a higher *CPP*_2024_ of 192 citations per publication.

Table 5 presents the publication characteristics of the 12 most productive institutions with at least 13 highly cited articles. Among them, institutions from the USA were most prominent, represented by nine institutions, followed by one institution each from Canada, Germany, and Italy, respectively. Only four of the top ten institutions had published single-author articles.

Allergan Pharmaceut Inc (USA) led in three of the six publication indicators:

1. Total publications (*TPs*): A total of 38 highly cited articles (5.9% of 641 highly cited articles);
2. Institutionally collaborative publications (*CP*_Is_): A total of 35 articles (8.1% of 431 inter-institutionally collaborative articles);
3. Single-author publications (*SPs*): Two articles (6.2% of 32 single-author articles).

Additionally, the Deutsche Klinik für Diagnostik (Germany) and the Buddhist Tzu Chi General Hospital (Taiwan) also led in single-author publications (*SPs*), each with two single-author articles.

The Baylor College of Medicine (USA) ranked at the top in the other three publication indicators:

1. Independent publications (*IP*_Is_): A total of 10 articles (4.8% of 210 inter-institutionally collaborative articles);
2. First-author publications (*FPs*): A total of 15 articles (2.3% of 641 first-author articles);
3. Corresponding-author publications (*RPs*): A total of 15 articles (2.5% of 609 corresponding-author articles).

Among the top 12 institutions, the University of California Irvine (USA), with a *TP* of 14 articles, had the highest *CPP*_2024_ of 303 citations per publication for all highly cited articles. Allergan Pharmaceut Inc (USA) had the highest *CPP*_2024_ of 344 and 277 citations per publication for single-institution articles and single-author articles, respectively. The University of Padua (Italy), with an *FP* of six articles and an *RP* of four articles, had the highest *CPP*_2024_ of 385 and 524 citations per publication for first-author articles and corresponding-author articles, respectively. The University of California San Diego (USA), with a *CP*_I_ of nine articles, had the highest *CPP*_2024_ of 381 citations per publication for inter-institutionally collaborative articles.

As shown in Table 5, the top institutions contributing to highly cited botulinum toxin research vary not only in publication volume but also in citation impact per article. The University of California Irvine recorded the highest citations per publication (*CPP*_2024_ = 303 citations), and the University of Padua in Italy demonstrated similarly strong performance, achieving the highest *CPP*_2024_ for both first-author (385 citations) and corresponding-author (524 citations) articles.

Allergan Pharmaceutical Inc., the developer of onabotulinumtoxinA (Botox), stood out with the highest *CPP*_2024_ values across all authorship and collaboration types, including single-institution (344 citations) and single-author (277 citations) articles, despite not being an academic institution. Additionally, the University of California San Diego (*CPP*_2024_ = 381 citations for collaborative articles), along with institutions such as McGill University (Canada) and Hannover Medical School (Germany), demonstrated that inter-institutional collaboration may further amplify citation impact. Together, the trends illustrated in Table 5 highlight how scholarly visibility in botulinum toxin research is shaped by institutional focus, author roles, and the interplay between academia and industry.

For 643 highly cited botulinum toxin-related articles, the *APP* was recorded at 6.2 authors per publications, with a maximum of 56 authors in an article titled “The 2018 ISDE achalasia guidelines” published in 2018 [31]. Zaninotto et al. (2018) authored the article, boasting 13 countries and 42 institutions [31]. A total of 641 of 643 highly cited articles had information about corresponding countries in the SCI-EXPANDED database. Only five highly cited articles were published by multiple corresponding authors. Of the 643 highly cited botulinum toxin-related articles, 63% articles were published by groups of two to six authors, including 106 highly cited articles (16% of 643 articles), 86 articles (13%), 78 articles (12%), 67 articles (10%), and 66 articles (10%) that were written by groups of five, six, four, three, and two authors, respectively. Table 6 lists the top 16 productive authors with three publication indicators, their citation indicators, and *Y*-index constants [32]. J. Jankovic is the most productive author, boasting an impressive 22 highly cited articles, among which six were authored as the first author and eight as the corresponding author. M. Naumann published the top seven highly cited first-author articles and the top eight corresponding-author articles. Compared to the top 16 authors in Table 6, E.R. Chapman, with a *TP* of 10 articles and an *FP* of one article, had the highest *CPP*_2024_ of 367 and 268 citations per publication for total articles and first-author articles, respectively. K.R. Aoki, with an *RP* of three articles, had the highest *CPP*_2024_ of 279 citations per publication for corresponding-author articles.

Eight of the 16 productive authors, including M. Naumann, J. Jankovic, S.K. Aurora, K.R. Aoki, J.O. Dolly, A. Blitzer, M.F. Brin, and S.D. Silberstein, were also found to be the top 16 publication potential authors in cochlear implants research as evaluated by *Y*-index.

A total of 442 (69% of 643) highly cited botulinum toxin-related articles in SCI-EXPANDED included both first- and corresponding-author information and were used to calculate the *Y*-index for individual authors. These articles were contributed by 2247 unique authors. Among them, 1788 authors (80%) did not contribute as either first or corresponding author and were therefore assigned a *Y*-index of (0, 0). For example, O. Rossetto and E.A. Johnson authored 10 highly cited articles but had a *Y*-index of (0, 0), indicating no first- or corresponding-author role. A total of 93 authors (4.1%) published only as corresponding authors, with *h* = π/2. One such example is J.D. Marks, who had a *Y*-index of (4, π/2) based on two corresponding-author articles. Twenty-five authors (1.1%) published a greater number of corresponding-author articles than first-author articles (π/2 > *h* > π/4) with *FP* > 0. Meanwhile, 210 authors (9.3%) had equal numbers of first- and corresponding-author articles, resulting in *h* = π/4. Only five authors (0.22%), including R.B. Lipton, C.M. Shaari, H.G. Gassner, L. Restani, and M.A. Khashab, had a *Y*-index of (3, 0.4636), indicating more first-author articles than corresponding-author articles (π/4 > *h* > 0) with *RP* > 0. In contrast, 126 authors (5.6%) published only first-author articles (*h* = 0).

**Figure 3:**
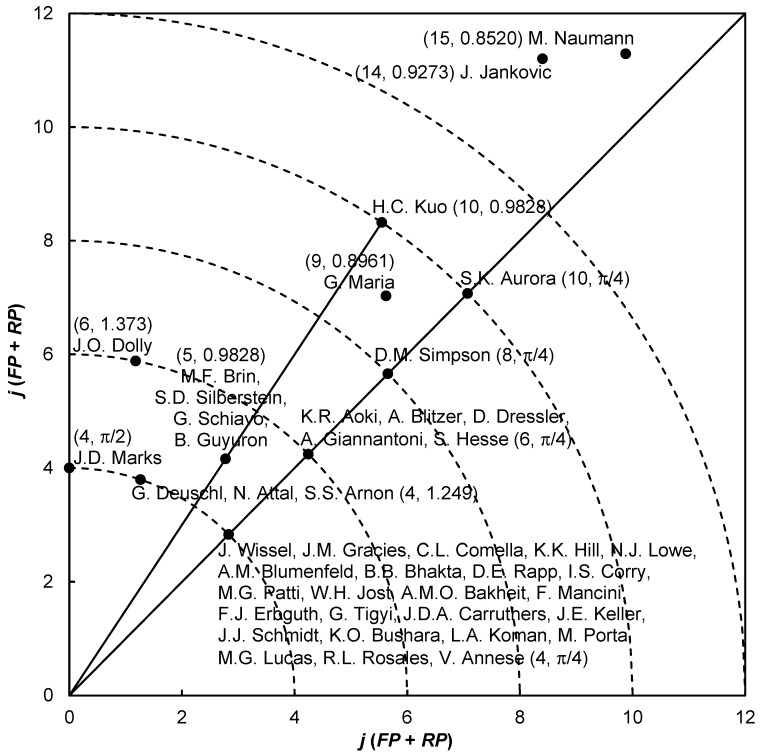
Distribution of the top 44 authors with *Y*-index in botulinum toxin research field.

Figure 3 illustrates the distribution of *Y*-indices for the 44 highly cited authors with a *j* of 4 or more. Each point on the plot represents a unique *Y*-index value that may correspond to one or multiple authors. For instance, J. Wissel and 23 other authors shared the same *Y*-index of (4, π/4), reflecting identical publication potential and characteristics. M. Naumann, with a *Y*-index of (15, 0.8520), had the highest publication potential (*j* = 15), followed by J. Jankovic (with a *j* of 14).

Authors such as J.D. Marks (4, π/2), G. Deuschl, N. Attal, S.S. Arnon (4, 1.249), J. Wissel, and 23 other authors (4, π/4) had equal *j* values but different *h* values, indicating the same publication potential but different authorship roles, only corresponding-author articles for Marks, more corresponding-author articles for Deuschl, Attal, and Arnon, and the same number of first-author articles and corresponding-author articles for Wissel and 23 other authors. These authors lie on the same *j* = 4 curve in Figure 3 but at different angular positions due to differences in *h*. Similarly, J.O. Dolly (6, 1.373), K.R. Aoki, A. Blitzer, D. Dressler, A. Giannantoni, and S. Hesse (6, π/4) all lie on the *j* = 6 curve, with Dolly publishing more corresponding-author articles than first-author articles and Aoki, Blitzer, Dressler, Giannantoni, and Hesse publishing an equal number of first- and corresponding-author articles.

Authors along the diagonal line (*h* = π/4), such as S.K. Aurora (10, π/4), D.M. Simpson (8, π/4), K.R. Aoki, and four other authors (6, π/4) and J. Wissel and 23 other authors (4, π/4), all had equal contributions as first and corresponding authors but differed in overall publication potential (*j*) in the following order: Aurora with a *j* of 10, Simpson (*j* = 8), Aoki and four other authors (*j* = 6), and Wissel and 23 other authors (*j* = 4). Similarly, H.C. Kuo (10, 0.9828) and M.F. Brin and three other authors (5, 0.9828) were located on a straight line (*h* = 0.9828). They have the same publication characteristics. However, Kuo had higher publication potential, with a *j* of 10; Brin followed and then three other authors with a *j* of 5.

In summary, the distribution of authors in Figure 3 reflects distinct families of publication characteristics (as indicated by *h*) and publication potential (as indicated by *j*). Authors with the same *j* value lie on the same curve, while those with the same *h* value fall along the same angular direction from the origin.

### 3.5. The Most Frequently Cited Articles and the Most Impactful Articles in 2024

**Figure 4:**
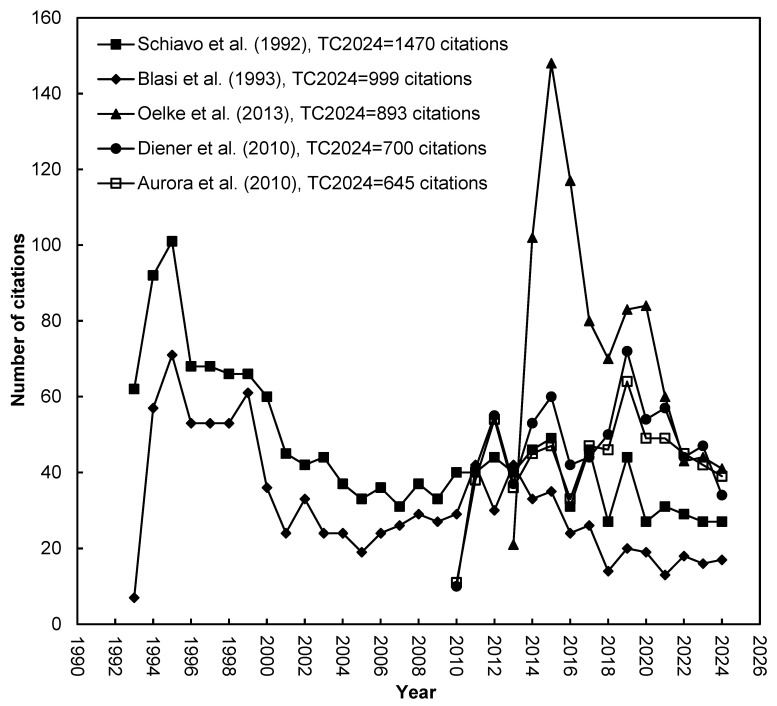
Citation histories of the top five articles with search keywords in their title or author keywords [33,34,35,36,37].

**Figure 5:**
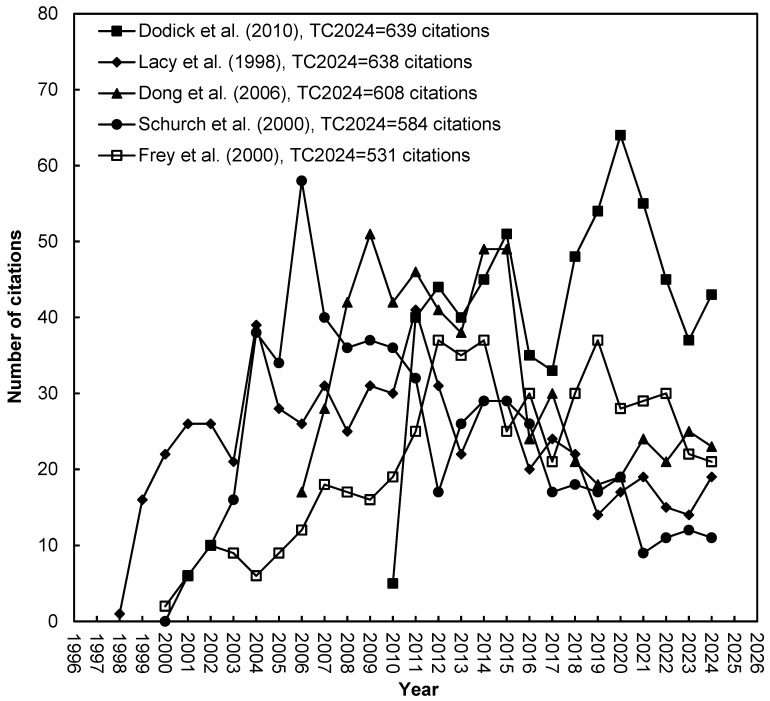
Citation histories of the top 6–10 articles with search keywords in their title or author keywords [38,39,40,41,42].

Table 7 provides a detailed list of the top ten most frequently cited articles that include search keywords either in the title or author keywords. Seven of the top ten articles originated from the USA, followed by Germany (five articles), France (three), Switzerland (three), and one article each from Denmark, Greece, Italy, Netherlands, Spain, and the UK, respectively. The citation histories of these 10 articles are depicted in Figure 4 and Figure 5.

Five of the articles were ranked in the top ten of *C*_2024_ and *TC*_2024_. These articles are not only highly cited articles but also the most impactful in the most recent year of 2024.

#### 3.5.1. “Tetanus and Botulinum-B Neurotoxins Block Neurotransmitter Release by Proteolytic Cleavage of Synaptobrevin” (Schiavo et al., 1992) [33]

This article was published by seven authors from Italy, France, and the USA and had a *TC*_2024_ of 1470 citations (ranked first) and a *C*_2024_ of 27 citations (ranked eighth).

The seminal paper by Schiavo et al. (1992) [33], published in *Nature*, revealed a groundbreaking mechanism by which the tetanus neurotoxin (TeTx) and botulinum neurotoxin type B (BoNT/B) inhibit neurotransmitter release. The authors demonstrated that these clostridial neurotoxins act as zinc-dependent endopeptidases that specifically cleave synaptobrevin-2 (SYB-2), an integral membrane protein of small synaptic vesicles, at a single peptide bond (Gln76–Phe77). This cleavage disrupts the vesicle fusion process essential for neurotransmitter exocytosis, thereby effectively blocking synaptic transmission. The study used purified synaptic vesicles from rat brain to establish the specificity of proteolytic activity. The results showed that while SYB-2 is cleaved by TeTx and BoNT/B, its isoform SYB-1 remains resistant due to a valine residue at the cleavage site. The proteolytic activity was inhibited by EDTA and captopril, confirming the role of zinc in enzymatic function. Furthermore, synthetic peptides mimicking the SYB-2 cleavage site successfully delayed the onset of neurotoxicity in Aplysia neurons, providing functional evidence of SYB-2’s central role in neurotransmitter release.

#### 3.5.2. EAU Guidelines on the Treatment and Follow-Up of Non-Neurogenic Male Lower Urinary Tract Symptoms Including Benign Prostatic Obstruction (Oelke et al., 2013) [35]

The article published by nine authors from Germany, Switzerland, France, the UK, Greece, Denmark, and the Netherlands with a *TC*_2024_ of 893 citations (ranked 3rd) and a *C*_2024_ of 41 citations (ranked 3rd). The 2013 European Association of Urology (EAU) guidelines by Oelke et al., published in *European Urology*, represent a comprehensive, evidence-based framework for managing non-neurogenic male lower urinary tract symptoms (LUTS), including benign prostatic obstruction (BPO). These guidelines, including key recommendations, synthesize data from decades of clinical studies, aiming to tailor management to symptom severity, underlying pathology, and patient preferences. Treatments span from conservative approaches like lifestyle modification to pharmacological therapies (including α1-blockers, 5α-reductase inhibitors, phosphodiesterase type 5 inhibitors, antimuscarinics, and desmopressin) and surgical interventions such as TURP, laser therapies, and minimally invasive techniques. It also includes a visual synthesis that reinforces the guideline’s core perspective.

#### 3.5.3. “OnabotulinumtoxinA for Treatment of Chronic Migraine: Results from the Double-Blind, Randomized, Placebo-Controlled Phase of the PREEMPT 2 Trial” (Diener et al., 2010) [36]

This article was published by eight authors from Germany and the USA and had a *TC*_2024_ of 700 citations (ranked fourth) and a *C*_2024_ of 34 citations (ranked fifth). The PREEMPT 2 trial, published by Diener et al. in Cephalalgia (2010) [36], is a landmark phase 3, randomized, double-blind, placebo-controlled study evaluating the efficacy and safety of onabotulinumtoxinA (BOTOX^®^) in adults with chronic migraine (CM). This condition, defined as experiencing headaches on ≥15 days per month (of which ≥8 are migrainous), affects up to 2.4% of the population and significantly impairs quality of life. PREEMPT 2 enrolled 705 patients who received two cycles of either onabotulinumtoxinA (155–195 U) or placebo injections over 24 weeks. The study’s primary endpoint, e.g., mean change in headache days per 28-day cycle from baseline to week 24, was met with a statistically significant reduction in the active treatment group (−9.0 days) compared to placebo (−6.7 days, *p* < 0.001). OnabotulinumtoxinA also showed superiority across all five ranked secondary endpoints: reductions in migraine days, moderate/severe headache days, cumulative headache hours, HIT-6 disability scores, and headache episodes. Notably, the treatment group experienced a substantial improvement in quality of life and functioning as measured by MSQ and HIS scores. Figure 4 of the article visualizes the treatment’s primary outcome, clearly demonstrating the sustained reduction in headache frequency over time in the active arm relative to placebo.

#### 3.5.4. “OnabotulinumtoxinA for Treatment of Chronic Migraine: Results from the Double-Blind, Randomized, Placebo-Controlled Phase of the PREEMPT 1 Trial” (Aurora et al., 2010) [37]

This article was published by eight authors from the USA and Germany and had a *TC*_2024_ of 645 citations (ranked fifth) and a *C*_2024_ of 39 citations (ranked fourth). This article, published by Aurora et al. in *Cephalalgia* (2010) [37], was the first of two pivotal phase 3 studies investigating the efficacy and safety of onabotulinumtoxinA (BOTOX^®^) as prophylactic treatment for CM. Conducted across 56 centers in North America, the study enrolled 679 adults with CM, characterized by ≥15 headache days per month, of which ≥8 were migrainous. Patients received two cycles of onabotulinumtoxinA (155–195 U) or placebo injections over a 24-week double-blind phase. The primary endpoint, which was mean change in the frequency of headache episodes, did not significantly differ between treatment and placebo groups (−5.2 vs. −5.3; *p* = 0.344). However, significant treatment benefits were observed in several secondary endpoints. They were: (a) reduction in headache days (−7.8 vs. −6.4; *p* = 0.006); (b) reduction in migraine days (−7.6 vs. −6.1; *p* = 0.002); (c) reduced cumulative headache hours and frequency of moderate/severe headache days; and (d) improved health-related quality of life (HRQoL) via HIT-6 and MSQ v2.1 scores.

#### 3.5.5. “OnabotulinumtoxinA for Treatment of Chronic Migraine: Pooled Results from the Double-Blind, Randomized, Placebo-Controlled Phases of the PREEMPT Clinical Program” (Dodick et al., 2010) [38]

This article was published by eight authors from the USA and Germany and had a *TC*_2024_ of 639 citations (ranked sixth) and a *C*_2024_ of 43 citations (ranked second). Finally, Dodick et al.’s pooled analysis of the PREEMPT 1 and 2 clinical trials, published in *Headache* (2010), presents the most comprehensive evidence to date for the efficacy, safety, and tolerability of onabotulinumtoxinA (BOTOX^®^) as prophylactic treatment for CM. This study integrated results from two nearly identical randomized, double-blind, placebo-controlled phase 3 trials, encompassing 1384 patients. It was designed to increase statistical power, confirm the consistency of individual trial outcomes, and provide a broader understanding of the clinical utility of onabotulinumtoxinA. The primary endpoint, mean change from baseline in headache days over 24 weeks, showed a significant reduction in the onabotulinumtoxinA group compared to placebo (−8.4 vs. −6.6 days; *p* < 0.001). Secondary endpoints also favored onabotulinumtoxinA, including reductions in days with migraine, days with moderate or severe headache, days with cumulative headache hours, frequency of headache and migraine episodes, headache-related disability (HIT-6 score), and use of triptans. The proportion of patients achieving ≥50% reduction in headache days was also significantly higher in the treatment group (47.1% vs. 35.1%, *p* < 0.001).

### 3.6. Analysis of Words in Highly Cited Article Title and Author Keywords

Table 8 presents the 20 most frequently used words in the titles and author keywords of highly cited articles in the botulinum toxin research, excluding the predefined search keywords. From these 20 most frequently used words in the titles and author keywords, five main highly cited topics arise. They are: *botulinum toxin and spasticity*; *botulinum toxin in dystonia and movement disorders*; *botulinum toxin for cosmetic applications*; *neurogenic bladder and urinary disorders*; and *pain management and chronic conditions*. Below follows a brief discussion on each topic, including the most important words used in the titles and author keywords.

## 4. Discussion

The most cited article identified (Finnerup et al., 2015) exemplifies how systematic reviews with robust methodology can transcend specific therapeutic boundaries [29]. Even though “botulinum toxin A” was only mentioned in the abstract, this comprehensive review evaluated the efficacy and safety of various pharmacological treatments for neuropathic pain, synthesizing evidence from 229 randomized, double-blind, placebo-controlled trials. Although “botulinum toxin A” was only briefly mentioned in the abstract, the article’s extensive coverage of treatment modalities and its rigorous methodological approach positioned it as a seminal reference in the pain management literature. Its impact extends beyond the specific context of botulinum toxin, reflecting its value as a cornerstone resource for clinical decision-making and guideline development in neuropathic pain therapy. Beyond its scientific influence, this pattern may also reflect how highly cited evidence contributes to healthcare decision-making and the prioritization of treatment strategies across clinical settings. For clinicians, these findings highlight the most influential evidence shaping current therapeutic applications, particularly in areas such as chronic migraine, spasticity, and urological disorders. For policymakers, understanding the concentration of highly cited research may support prioritization of high-impact areas and resource allocation. In particular, widely cited studies may play a role in shaping guideline development and influencing the allocation of healthcare resources in areas with high disease burden, such as chronic pain and neurological disorders. From a clinical perspective, this influence is likely driven by the study’s robust evidence base and its relevance for interdisciplinary care, highlighting how evidence-based pharmacotherapy frameworks can shape the wider field of pain management [29]. The prominent role of industry-linked research, particularly from pharmaceutical companies such as Allergan, reflects the importance of translational pathways in botulinum toxin development. Industry involvement may facilitate clinical trials, regulatory approval, and implementation in clinical practice but may also influence research agendas and citation patterns. These findings should be interpreted in the context of citation dynamics, where older publications and large collaborative studies tend to accumulate more citations over time. Therefore, citation impact does not necessarily equate to methodological quality or clinical superiority but rather reflects visibility, dissemination, and integration into the scientific discourse.

When it comes to the accumulation of citations, it inherently requires time, and in the case of botulinum toxin research, this process is notably prolonged. Based on the results, it was observed that highly cited articles in this field took an average of 18 years to reach their citation peak, a duration longer than that seen in other medically related research areas. These findings suggest that highly cited botulinum toxin research has a relatively long citation life cycle, which may reflect both the gradual expansion of clinical indications and the sustained influence of landmark mechanistic and translational studies.

Even though the year 2005 was the most productive year when it comes to highly cited botulinum toxin-related papers, the most recent highly cited publication was published in 2022 by Ferrari et al. [30]. Although the title did not include a direct keyword, the abstract referenced “OnabotulinumtoxinA,” indicating its relevance to the field. This thorough primer provides an in-depth overview of migraine pathophysiology, diagnosis, epidemiology, and treatment strategies. Although no botulinum toxin-related terms appeared in the title, the abstract explicitly referenced “OnabotulinumtoxinA” as a preventive treatment option, confirming its relevance to botulinum toxin research. The article synthesizes current evidence on both pharmacological and non-pharmacological therapies, offering a global perspective on disease burden and management approaches. Its publication in a high-impact, widely read journal likely contributed to its rapid citation accumulation, reaching over 200 citations within just two years. From a profession’s perspective, this could be associated with both the clinical urgency of effective migraine management, including the growing clinical significance of botulinum toxin in migraine prevention and its integration into contemporary treatment guidelines.

Based on the facts that the papers with the highest citation impact (*CPP*_2024_) in the field of botulinum toxin research are international collaborations, as well as inter-institutional collaborations, this indicates that there is a greater possibility to produce papers with a strong outreach and impact in the scientific society when researchers and research groups collaborate. Thus, inter-institutional collaboration may further amplify citation impact through broader dissemination and cross-disciplinary appeal [43]. Further, among the top institutions contributing to highly cited botulinum toxin research, there are variations not only in publication volume but also in citation impact per article. Our findings reaffirm that international and inter-institutional collaboration correlates strongly with citation impact, supporting prior bibliometric evidence that multi-center authorship fosters higher scientific visibility and methodological rigor. These differences can be attributed to a combination of academic leadership, collaborative research structures, and alignment with industry-driven innovation. This is underscored by the finding that the University of California Irvine recorded the highest citations per publication, and the University of Padua demonstrated similarly strong performance in achieving the highest citations per publication for both first-author and corresponding-author articles. These findings reflect the exceptional influence of these institutions and underscores the importance of lead authorship roles for increasing research visibility. These observations are consistent with earlier bibliometric findings indicating that first- and corresponding-author positions are often associated with higher citation rates. In addition, on the other hand, corporate research seems to play a central role in the field of botulinum toxin. The developer of onabotulinumtoxinA (Botox), Allergan Pharmaceutical Inc., stood out with the highest citations per publication despite not being an academic institution. This remarkable impact likely stems from Allergan’s pivotal role in developing and commercializing one of the most widely studied botulinum toxin formulations which frequently serves as a reference standard in clinical research [44].

Several highly cited authors within this dataset have made distinct and long-standing contributions to the advancement of botulinum toxin research, often recognized beyond their publication metrics. Notably, D. Dressler is internationally renowned for his clinical and scientific work on the use of botulinum toxin in treating movement disorders, particularly dystonia. His research has been pivotal in defining treatment protocols and understanding long-term efficacy and resistance development, cementing his role as a clinical authority in the field [45,46]. K.R. Aoki, affiliated with Allergan Pharmaceutical Inc., has played a foundational role in elucidating the antinociceptive (pain-relieving) properties of botulinum toxin type A. His work expanded the therapeutic scope of onabotulinumtoxinA beyond muscular applications to include pain modulation, influencing FDA approvals for indications such as chronic migraine. Aoki’s position within the industry also highlights the importance of translational research and the interface between pharmacological innovation and clinical application [47,48]. J.O. Dolly has significantly contributed to the understanding of botulinum toxin pharmacokinetics and molecular mechanisms. His research has explored how the neurotoxin interacts with synaptic proteins and is internalized by neurons, advancing scientific knowledge of the drug’s mechanism of action at the cellular level [49]. These insights have informed safer and more targeted clinical use across both therapeutic and aesthetic domains.

These individuals not only rank among the most cited authors in this analysis but also exemplify how specific expertise, ranging from clinical application to molecular biology, can shape the evolution and reputation of botulinum toxin research over time.

Titles are often the first and sometimes the only element of an article encountered by readers, making them crucial for capturing attention and conveying key insights [50]. Author keywords, which are voluntarily selected by the authors, reflect the primary themes of the study and are typically not constrained by a controlled vocabulary [51].

When it comes to the five most impactful articles, one has to consider why they are so cited. When it comes to the article by Schiavo et al. from 1992 [33], one can suggest several compelling reasons to discuss why this article was the most cited in 2024. First, it provided a definitive molecular explanation for the neuroparalytic effects of tetanus and botulinum toxins, an insight that fundamentally advanced our understanding of synaptic transmission. By identifying synaptobrevin-2 as the critical target, the paper laid the groundwork for the SNARE hypothesis, which has since become a cornerstone of cellular membrane fusion studies. Second, the findings have had lasting clinical relevance. As the therapeutic use of botulinum neurotoxins expanded in neurology, pain management, and aesthetics, this study was increasingly cited to underpin the mechanistic rationale for such applications. The clear evidence of toxin specificity and inhibition also suggested novel therapeutic routes for treating tetanus and botulism, including the use of zinc endopeptidase inhibitors. Third, this article provided a visually compelling biochemical demonstration of the toxins’ mechanism of action. Such clarity helped the article maintain its pedagogical and experimental value across decades. Finally, the article regained prominence in 2024 amid a resurgence of interest in synaptic dysfunction in neurodegenerative diseases. Researchers investigating conditions like Alzheimer’s disease, Parkinson’s disease, and ALS frequently cite this work to contextualize disruptions in synaptic vesicle cycling and neurotransmitter release. In summary, the study by Schiavo et al. represents a pivotal contribution to neuroscience, offering both mechanistic precision and lasting translational impact. Its profound influence on research and clinical practice explains its status as one of the longitudinally most cited and impactful articles in the scientific literature of 2024. One the other hand, the importance of the article by Oelke et al. from 2013 [35] can most probably be explained by the significant resurgence in citations and influence in 2024. First, the aging global male population has led to a dramatic rise in LUTS prevalence, heightening demand for standardized treatment frameworks. Second, clinical trials and health policy discussions now increasingly emphasize evidence-based, individualized care, directly aligning with the methodology and grading system used in this guideline (e.g., Oxford Centre for Evidence-based Medicine levels). The clarity and hierarchy of its recommendations have made it a default reference in both academic and clinical urology. Third, the rising integration of shared decision-making in urological care has renewed interest in practical, patient-oriented algorithms such as those detailed in this document. The guideline’s broad scope includes both drug-naïve and previously treated patients, surgical thresholds, and long-term outcome data, further enhancing its applicability. Finally, increased scrutiny on overdiagnosis and overtreatment in urology has made this publication a cornerstone reference for defining conservative management standards, particularly around watchful waiting and lifestyle-based interventions. In summary, the EAU guidelines authored by Oelke et al. offer a robust, high-quality synthesis of urological evidence that continues to shape global practice. It shows a vast uptick in citations during 2014–2016, and after that, it reached a plateau among the most cited articles in this area, which reflects not only its foundational role in LUTS management but also its enduring relevance in a healthcare climate demanding both rigor and nuance. Further, when it comes to the article by Diener et al. from 2010 [36], Figure 4 in this article visualizes the treatment’s primary outcome, clearly demonstrating the sustained reduction in headache frequency over time in the active arm relative to placebo. This figure has become iconic in illustrating BOTOX’s therapeutic trajectory and has been heavily cited in subsequent migraine research and regulatory guidance. In 2024, the PREEMPT 2 trial resurfaced as one of the most cited studies due to several converging factors. First, chronic migraine has seen increasing global prevalence and awareness, partly due to long-term pandemic stress and digital screen exposure. Second, regulatory bodies and clinical guidelines, including those in the U.S. and EU, now recognize onabotulinumtoxinA as a standard preventive therapy, anchoring this publication as a reference point for clinical justification. Third, growing interest in multimodal migraine management and integration of biologics and neurotoxins in personalized medicine has brought renewed attention to foundational trials. Additionally, healthcare systems are prioritizing treatments that show both efficacy and cost-effectiveness in reducing disability, an area where onabotulinumtoxinA consistently performs well. The PREEMPT 2 dataset has also supported numerous post hoc analyses, meta-analyses, and real-world studies, further amplifying its reach. Importantly, this trial addressed a previously underserved patient population: those with medication overuse and refractory migraine who were often excluded from earlier studies. The study’s robust design, stringent endpoint adjudication, and high patient retention (≥89% completion) underscore its methodological quality and clinical relevance. In summary, the PREEMPT 2 trial is not only a pivotal efficacy study for onabotulinumtoxinA in chronic migraine but also a cornerstone in headache research methodology. Its widespread clinical uptake, regulatory influence, and data longevity explain its continued high level of citations and its continued impact on patient care over time. In addition, even though there was no significant effect regarding the primary episode-based endpoint in the article by Aurora et al. from 2010 [37], this article could show a significant treatment effect in reducing headache days over time. This result emphasizes the need to prioritize headache days over episodes in future CM trials, which is an insight that seems to have a lasting influence. In 2024, this article was among the most frequently cited due to renewed focus on chronic migraine management amid rising global prevalence, especially in younger adults and post-pandemic populations. Unlike PREEMPT 2, which met both primary and secondary endpoints, PREEMPT 1 gained retrospective significance for revealing methodological challenges in endpoint selection, placebo response, and baseline imbalances. These discussions have become increasingly relevant as new migraine biologics and delivery paradigms (e.g., long-acting injectables, wearable neuromodulation) undergo regulatory scrutiny using similar metrics. Furthermore, the study’s inclusion of patients with medication overuse and treatment-refractory migraine expanded its clinical relevance. Also, the present era with personalized medicine approaches, with, e.g., targeted biologics and neurotoxins, makes this article highly cited, since it provides foundational data justifying the use of onabotulinumtoxinA in real-world, high-disability cases. An additional reason for its position among the top-cited publications was its safety profile. Treatment-related adverse events, while more frequent than in the placebo arm (25.3% vs. 11.7%), were generally mild and consistent with known effects. Importantly, serious adverse events were rare and not treatment-related. In summary, while PREEMPT 1 did not achieve statistical superiority for its primary endpoint, its robust secondary findings, patient-centered outcomes, and design insights have had enduring impact. Its citation course already started strongly during its first year and has remained on a similar level throughout the years, which could reflect both the evolution of clinical trial methodology and the ongoing relevance of onabotulinumtoxinA in the expanding landscape of chronic migraine therapy. Finally, the article by Dodick et al. from 2010 [38] also has an illustration showing the superior efficacy of onabotulinumtoxinA across time, beginning at week 4 and persisting through week 24, offering a visual anchor for the treatment’s durable impact. This pooled study experienced a resurgence in influence in 2024 due to several critical developments. First, onabotulinumtoxinA remains the only approved therapy specifically indicated for chronic migraine, distinguishing it from newer but broader-acting CGRP monoclonal antibodies. As healthcare systems increasingly emphasize value-based care, the PREEMPT pooled analysis continues to serve as a gold standard for evidence of long-term, real-world efficacy and safety in a previously treatment-resistant population. Second, growing interest in precision neurology and patient-centered outcomes has led researchers and guideline developers to revisit data sources that include responder analyses, HRQoL measures, and functional improvements—areas in which this pooled study excels. For example, substantial improvement in MSQ domains and HIT-6 scores reinforce the drug’s impact beyond mere reduction in headache frequency. Third, with chronic migraine being newly prioritized in many national health systems due to its high societal and occupational burden, the PREEMPT pooled analysis gained renewed visibility as a model of comprehensive trial design. The study’s inclusion of patients with medication overuse and its stringent statistical design have made it a cornerstone for both clinical and regulatory frameworks. Finally, safety data revealed that most adverse events, such as neck pain, muscular weakness, and injection-site discomfort, were mild or moderate and transient; thus, no new safety signals emerged. In conclusion, the pooled analysis by Dodick et al. represents the most definitive evidence base for the preventive use of onabotulinumtoxinA in chronic migraine [38]. In its first year, it had already become one of the most cited articles (2011), and it has continued being highly cited, with a tendency to show a yearly increase; it is one of the most cited articles in this field since the year 2018 because it mirrors a combination of enduring clinical relevance, superior study design, and a growing focus on disability reduction and personalized therapy in neurological care.

The five main highly cited topics that arose were *botulinum toxin and spasticity*, *botulinum toxin in dystonia and movement disorders*, *botulinum toxin for cosmetic applications*, *neurogenic bladder and urinary disorders*, and *pain management and chronic conditions*. Below follows a brief discussion on each topic, including the most important words used in the titles and author keywords.

Regarding the topic “*botulinum toxin and spasticity*”, research in this area primarily focuses on the therapeutic use of botulinum toxin for reducing spasticity, particularly in patients with neurological conditions such as cerebral palsy and post-stroke complications. The most cited articles examine treatment efficacy, dosage optimization, and long-term outcomes. This topic reflects the central role of botulinum toxin in neuromuscular rehabilitation and functional improvement. This topic is based on these words in the titles and author keywords: *spasticity*, *cerebral palsy*, *urinary incontinence*, *muscle spasticity*.

When it comes to the topic “*botulinum toxin in dystonia and movement disorders*”, this cluster includes studies investigating botulinum toxin as a first-line treatment for various movement disorders, notably cervical dystonia, blepharospasm, and spasmodic torticollis. Chronic migraine is also prominent within this category, highlighting the toxin’s expanding neurological indications. The research often emphasizes clinical trial outcomes and mechanistic insights into symptom modulation. This topic is based on these words in the titles and author keywords: *dystonia*, *cervical dystonia*, *chronic migraine*, *blepharospasm*, *spasmodic torticollis*.

Regarding the topic “*botulinum toxin for cosmetic applications*”, research in this area primarily focuses on cosmetic use of botulinum toxin, especially for treating glabellar and forehead lines, and represents one of the most commercially visible areas of research. Highly cited articles address aesthetic outcomes, patient satisfaction, safety profiles, and dosing strategies. This theme illustrates the interdisciplinary nature of botulinum toxin research, intersecting dermatology, plastic surgery, and consumer health. This topic is based on these words in the titles and author keywords: *cosmetic purpose*, *glabellar lines*, *forehead lines*, *aesthetics*.

When it comes to “*neurogenic bladder and urinary disorders*”, this theme covers research evaluating botulinum toxin’s effectiveness in treating lower urinary tract dysfunctions such as detrusor overactivity and neurogenic bladder. Articles often discuss injection techniques, therapeutic durability, and quality-of-life improvements in patients with spinal cord injuries or multiple sclerosis. The topic underscores the growing urological relevance of botulinum toxin therapy. This topic is based on these words in the titles and author keywords: *urinary incontinence*, *neurogenic bladder*, *detrusor overactivity*, *bladder*, *urinary bladder*.

Finally, regarding the topic “*pain management and chronic conditions*”, botulinum toxin has been increasingly studied for its antinociceptive properties in chronic pain conditions, including chronic migraine, myofascial pain syndrome, and neuropathic pain. The literature emphasizes both preventive and acute treatment effects, as well as mechanisms underlying pain modulation. This topic highlights the shift toward broader therapeutic applications beyond traditional motor symptoms. This topic is based on these words in the titles and author keywords: *chronic migraine*, *pain*, *myofascial pain syndrome*, *acute treatment*, *prevention*.

Taken together, the word frequency analysis of article titles and author keywords reveals five dominant research themes within the highly cited literature on botulinum toxin. These themes span both therapeutic and aesthetic applications, illustrating the versatility and multidisciplinary relevance of the toxin. A significant portion of the literature focuses on its clinical use in treating spasticity, dystonia, and chronic pain—conditions often associated with substantial patient burden and unmet therapeutic needs. In parallel, cosmetic applications emerge as a commercially impactful and widely studied domain, while urological uses, especially for neurogenic bladder, highlight its expanding role in specialized care. The frequent appearance of terms such as “spasticity”, “dystonia”, “pain”, “urinary incontinence”, and “cosmetic” underscores a research landscape that is both clinically diverse and application-driven. Together, these findings suggest that highly cited research in this field is closely aligned with areas of high clinical demand, regulatory expansion, and patient-centered outcomes, reinforcing botulinum toxin’s position as a widely adopted and continuously evolving therapeutic agent. From a healthcare perspective, these findings highlight the importance of aligning research priorities with clinical needs and real-world treatment demands. A better understanding of influential research patterns may support evidence-based practice, facilitate interdisciplinary collaboration, and contribute to more effective implementation of botulinum toxin therapies across healthcare systems.

Across all five thematic domains, highly cited research suggests a consistent convergence of mechanistic depth and clinical translation. Recent bibliometric analyses in other therapeutic toxin fields suggest that long-term impact depends more on interdisciplinary applicability than on initial citation velocity [12]. This reinforces the view that botulinum toxin research thrives at the intersection of neurology, rehabilitation, and aesthetics, with continued expansion into pain and urology.

### 4.1. Study Strengths

This study has several strengths. First, it is based on a comprehensive dataset derived from the Web of Science Core Collection (SCI-EXPANDED), using a reproducible and well-defined search strategy. Second, the application of standardized bibliometric indicators, including *TC*_2024_ and the *Y*-index, allowed for consistent and comparable evaluation of publication performance. Third, the focus on highly cited articles enabled the identification of influential research shaping clinical practice and therapeutic development across multiple healthcare domains, including neurology, pain management, urology, and aesthetic medicine.

### 4.2. Study Limitations

This study has several limitations that should be considered. The analysis was restricted to the Web of Science Core Collection (SCI-EXPANDED), which may not capture all relevant publications indexed in other databases such as Scopus or PubMed. The use of a citation threshold (*TC*_2024_ ≥ 100) may favor older publications that have had more time to accumulate citations, potentially underrepresenting more recent but clinically relevant studies. Citation counts do not necessarily reflect clinical effectiveness or quality of evidence, as they may also be influenced by factors such as journal visibility, research trends, and self-citation practices. Furthermore, although efforts were made to standardize affiliations and keywords, variations in indexing and terminology may have affected data consistency. The use of topic (TS) searches, which include *Keywords Plus*, may capture marginally related publications, and although the “front page” filter was applied to improve relevance, this approach may still introduce selection bias. Therefore, the findings of this study should be interpreted as indicators of scientific influence rather than direct measures of methodological quality or clinical effectiveness.

## 5. Conclusions

This bibliometric analysis provides insight into influential research patterns in the botulinum toxin literature. By identifying highly cited articles, key institutions, prolific authors, and emerging themes, the study highlights the multidimensional nature of botulinum toxin research, spanning neurology, urology, dermatology, and aesthetics. The strong presence of industry-linked research, particularly from Allergan, underscores the significant role of commercial innovation in shaping scholarly impact. The findings also emphasize the value of collaborative authorship and first-/corresponding-author contributions in achieving higher citation performance. By mapping thematic clusters and author productivity through indicators like the *Y*-index, the study offers a structured perspective on research influence. These insights may guide future investigations, foster strategic collaborations, and inform policy decisions in both clinical and research environments. However, the findings should be interpreted as descriptive rather than causal, and conclusions regarding clinical integration or impact should be formed with caution.

## Figures and Tables

**Table 1:** Citations and authors according to the document type.

Document Type	*TP*	%	*AU*	*APP*	*TC*_2024_	*CPP*_2024_
Article	643	79	3992	6.2	118,175	184
Review	162	20	883	5.5	32,888	203
Proceedings paper	48	5.9	198	4.1	7735	161
Note	8	1.0	42	5.3	1513	189
Book chapter	6	0.73	9	1.5	1023	171
Editorial material	2	0.24	6	3.0	260	130
Letter	2	0.24	6	3.0	270	135
Retracted publication	1	0.12	2	2.0	123	123

The summed total exceeds the number of unique publications since Web of Science document types may overlap. *TP*: total number of publications; %: percentage of articles in all articles; *AU*: total number of authors; *APP*: average number of authors per publication; *TC*_2024_: total number of citations from WoSCC since publication year until the end of 2024; *CPP*_2024_: average number of citations per publication (*TC*_2024_/*TP*).

**Table 2:** The top 10 most productive Web of Science categories in SCI-EXPANDED.

Web of Science Category	No. *J*	*TP* (%)	*APP*	*CPP*_2024_
Clinical neurology	211	187 (29)	6.2	184
Neurosciences	270	107 (17)	4.9	186
Surgery	212	84 (13)	5.8	142
Biochemistry and molecular biology	285	71 (11)	6.2	189
Urology and nephrology	89	61 (9.5)	6.4	192
Multidisciplinary sciences	72	34 (5.3)	7.7	260
Cell biology	191	33 (5.1)	6.5	221
Dermatology	69	26 (4.0)	3.8	152
Gastroenterology and hepatology	93	26 (4.0)	10	169
General and internal medicine	167	25 (3.9)	6.8	245

No. *J*: number of journals in a category in 2023; *TP*: total number of articles; *APP*: average number of authors per publication; *CPP*_2024_: average number of citations per publication (*TC*_2024_/*TP*).

**Table 3:** The top ten most productive journals.

Journal	*TP* (%)	*IF*_2023_	*APP*	*CPP*_2024_	*Web of Science Category*
*Neurology*	37 (5.8)	7.7	6.9	191	Clinical neurology
*Journal of Urology*	25 (3.9)	5.9	6.6	218	Urology and nephrology
*Journal of Biological Chemistry*	23 (3.6)	4.0	5.5	195	Biochemistry and molecular biology
*Movement Disorders*	20 (3.1)	7.4	5.6	144	Clinical neurology
*Journal of Neurology Neurosurgery and Psychiatry*	20 (3.1)	8.7	7.1	146	Clinical neurology Psychiatry Surgery
*European Urology*	19 (3)	25.3	7.7	202	Urology and nephrology
*Plastic and Reconstructive Surgery*	18 (2.8)	3.2	3.8	143	Surgery
*Journal of Neuroscience*	16 (2.5)	4.4	5.3	190	Neurosciences
*Headache*	15 (2.3)	5.4	4.6	240	Clinical neurology
*Proceedings of the National Academy of Sciences of the United States of America*	15 (2.3)	9.4	6.5	196	Multidisciplinary sciences

*TP*: total number of articles; %: percentage of articles in all articles; *IF*_2023_: journal impact factor in 2023; *APP*: average number of authors per publication; *CPP*_2024_: average number of per publication (*TC*_2024_/*TP*).

**Table 4:** Top 10 productive countries.

Country	*TP*	*TP* (n = 641)		*IP*_C_ (n = 462)		*CP*_C_ (n = 179)		*FP* (n = 641)		*RP* (n = 629)		*SP* (n = 32)	
		*R* (%)	*CPP*_2024_	*R* (%)	*CPP*_2024_	*R* (%)	*CPP*_2024_	*R* (%)	*CPP*_2024_	*R* (%)	*CPP*_2024_	*R* (%)	*CPP*_2024_
USA	347	1 (54)	202	1 (52)	181	1 (60)	251	1 (47)	187	1 (48)	186	1 (53)	176
Germany	121	2 (19)	219	2 (9.3)	142	2 (44)	261	2 (11)	182	2 (11)	160	2 (16)	133
UK	89	3 (14)	222	3 (7.8)	163	3 (30)	262	3 (9.0)	179	3 (9.2)	178	2 (16)	127
Italy	57	4 (8.9)	189	4 (6.7)	151	6 (15)	234	4 (5.9)	190	4 (5.9)	192	5 (3.1)	182
Canada	47	5 (7.3)	232	5 (3.9)	182	4 (16)	262	5 (3.4)	175	5 (3.3)	172	5 (3.1)	555
France	43	6 (6.7)	278	6 (3.5)	147	5 (15)	356	7 (3.1)	145	7 (3.2)	242	N/A	N/A
Australia	31	7 (4.8)	246	8 (3.2)	183	8 (8.9)	304	5 (3.4)	166	5 (3.3)	168	N/A	N/A
Switzerland	27	8 (4.2)	265	13 (0.65)	294	7 (13)	262	9 (2.8)	259	9 (2.5)	272	N/A	N/A
Japan	26	9 (4.1)	178	6 (3.5)	164	12 (5.6)	200	8 (3.0)	170	8 (3.0)	170	N/A	N/A
Belgium	19	10 (3.0)	199	13 (0.65)	155	8 (8.9)	207	17 (0.62)	162	17 (0.64)	162	N/A	N/A

*TP*: number of total articles; *TP R* (%): total number of articles and the percentage of total articles; *IP*_C_ *R* (%): rank and percentage of single-country articles in all single-country articles; *CP*_C_ *R* (%): rank and percentage of internationally collaborative articles in all internationally collaborative articles; *FP R* (%): rank and the percentage of first-author articles in all first-author articles; *RP R* (%): rank and the percentage of corresponding-author articles in all corresponding-author articles; *SP R* (%): rank and the percentage of single-author articles in all single-author articles; *CPP*_2024_: average number of citations per publication (*CPP*_2024_ = *TC*_2024_/*TP*); N/A: not available.

**Table 5:** Top 12 most productive institutions with at least 13 highly cited articles.

Institution	*TP*	*TP* (n = 641)		*IP*_I_ (n = 210)		*CP*_I_ (n = 431)		*FP* (n = 641)		*RP* (n = 609)		*SP* (n = 32)	
		*R* (%)	*CPP*_2024_	*R* (%)	*CPP*_2024_	*R* (%)	*CPP*_2024_	*R* (%)	*CPP*_2024_	*R* (%)	*CPP*_2024_	*R* (%)	*CPP*_2024_
API	38	1 (5.9)	227	8 (1.4)	344	1 (8.1)	217	10 (0.94)	273	4 (1.1)	258	1 (6.3)	277
BCM	28	2 (4.4)	183	1 (4.8)	172	2 (4.2)	189	1 (2.3)	159	1 (2.5)	159	N/A	N/A
U Wisconsin	18	3 (2.8)	298	8 (1.4)	145	4 (3.5)	329	5 (1.1)	254	4 (1.1)	254	N/A	N/A
Univ Padua	18	3 (2.8)	247	41 (0.48)	209	3 (3.9)	249	10 (0.94)	385	23 (0.66)	524	N/A	N/A
UCSF	17	5 (2.7)	212	3 (2.4)	116	7 (2.8)	252	2 (2.0)	169	2 (2.1)	169	4 (3.1)	107
Stanford Univ	16	6 (2.5)	260	8 (1.4)	295	6 (3.0)	252	5 (1.1)	210	8 (1.0)	214	N/A	N/A
UC San Diego	15	7 (2.3)	295	2 (2.9)	166	13 (2.1)	381	3 (1.2)	174	3 (1.3)	174	4 (3.1)	107
MSMC	15	7 (2.3)	192	8 (1.4)	146	7 (2.8)	204	3 (1.2)	215	8 (1.0)	188	4 (3.1)	208
UC Irvine	14	9 (2.2)	303	N/A	N/A	5 (3.2)	303	111 (0.16)	120	106 (0.16)	120	N/A	N/A
UBC	13	10 (2.0)	182	3 (2.4)	198	18 (1.9)	172	10 (0.94)	191	15 (0.82)	198	N/A	N/A
Harvard Univ	13	10 (2.0)	162	18 (1.0)	210	10 (2.6)	154	10 (0.94)	152	15 (0.82)	153	N/A	N/A
MHH	13	10 (2.0)	232	8 (1.4)	170	12 (2.3)	251	5 (1.1)	290	8 (1.0)	190	N/A	N/A

*TP*: total number of articles; *TP R* (%): total number of articles and percentage of total articles; *IP*_I_ *R* (%): rank and percentage of single-institution articles in all single-institution articles; *CP*_I_ *R* (%): rank and percentage of inter-institutionally collaborative articles in all inter-institutionally collaborative articles; *FP R* (%): rank and percentage of first-author articles in all first-author articles; *RP R* (%): rank and percentage of corresponding-author articles in all corresponding-author articles; *SP R* (%): rank and the percentage of single-author articles in all single-author articles; *CPP*_2024_: average number of citations per publication (*CPP*_2024_ = *TC*_2024_/*TP*); N/A: not available. API: Allergan Pharmaceut Inc, USA; BCM: Baylor College of Medicine, USA; U Wisconsin: University of Wisconsin, USA; Univ Padua: University of Padua, Italy; UCSF: University of California San Francisco, USA; Stanford Univ: Stanford University, USA; UC San Diego: University of California San Diego, USA; MSMC: Mount Sinai Medical Center, USA; UC Irvine: University of California Irvine, USA; UBC: University of British Columbia, Canada; Harvard Univ: Harvard University, USA; MHH: Hannover Medical School, Germany.

**Table 6:** Top 16 productive authors with ten highly cited articles or more.

Author	*TP* (n = 643)		*FP* (n = 643)		*RP* (n = 442)		*h* (n = 442)	Rank (*j*)
	Rank (*TP*)	*CPP*_2024_	Rank (*FP*)	*CPP*_2024_	Rank (*RP*)	*CPP*_2024_		
J. Jankovic	1 (22)	193	2 (6)	206	1 (8)	183	0.9273	2 (14)
M.F. Brin	2 (21)	257	7 (4)	193	9 (3)	173	0.9828	13 (5)
M. Naumann	3 (14)	179	1 (7)	141	1 (8)	142	0.8520	1 (15)
S.D. Silberstein	4 (13)	261	16 (3)	125	9 (3)	156	0.9828	13 (5)
C. Montecucco	5 (12)	275	84 (1)	209	69 (1)	209	π/4	69 (2)
H.K. Graham	6 (11)	197	84 (1)	121	69 (1)	121	π/4	69 (2)
K.R. Aoki	6 (11)	259	7 (4)	253	9 (3)	279	π/4	7 (6)
S.K. Aurora	6 (11)	282	4 (5)	247	4 (5)	247	π/4	3 (10)
L.A. Smith	9 (10)	179	N/A	N/A	69 (1)	110	π/2	262 (1)
O. Rossetto	9 (10)	302	N/A	N/A	N/A	N/A	0	460 (0)
W. Poewe	9 (10)	174	84 (1)	169	69 (1)	158	π/4	69 (2)
E.A. Johnson	9 (10)	220	N/A	N/A	N/A	N/A	0	460 (0)
J.O. Dolly	9 (10)	207	84 (1)	159	4 (5)	154	1.373	7 (6)
E.R. Chapman	9 (10)	367	84 (1)	268	27 (2)	233	1.107	45 (3)
A. Blitzer	9 (10)	183	2 (6)	146	9 (3)	166	π/4	7 (6)
T. Binz	9 (10)	301	N/A	N/A	69 (1)	177	π/2	262 (1)

*TP*: total number of highly cited articles; *FP*: first-author highly cited article; *RP*: corresponding-author highly cited article; *CPP*_2024_: average number of citations per publication (*CPP*_2024_ = *TC*_2024_/*TP*); *j*: a *Y*-index constant related to the publication potential; *h*: a *Y*-index constant related to the publication characteristics; N/A: not available.

**Table 7:** Top ten most frequently cited articles with search keywords in their title or author keywords.

Rank (*TC*_2024_)	Rank (*C*_2024_)	Title	Country	Reference
1 (1470)	8 (27)	Tetanus and botulinum-B neurotoxins block neurotransmitter release by proteolytic cleavage of synaptobrevin	Italy, France, USA	Schiavo et al. (1992) [33]
2 (999)	27 (17)	Botulinum neurotoxin-A selectively cleaves the synaptic protein SNAP-25	Germany, USA, Spain	Blasi et al. (1993) [34]
3 (893)	3 (41)	EAU guidelines on the treatment and follow-up of non-neurogenic male lower urinary tract symptoms including benign prostatic obstruction	Germany, Switzerland, France, UK, Greece, Denmark, Netherlands	Oelke et al. (2013) [35]
4 (700)	5 (34)	OnabotulinumtoxinA for treatment of chronic migraine: Results from the double-blind, randomized, placebo-controlled phase of the PREEMPT 2 trial	Germany, USA	Diener et al. (2010) [36]
5 (645)	4 (39)	OnabotulinumtoxinA for treatment of chronic migraine: Results from the double-blind, randomized, placebo-controlled phase of the PREEMPT 1 trial	USA, Germany	Aurora et al. (2010) [37]
6 (639)	2 (43)	OnabotulinumtoxinA for treatment of chronic migraine: Pooled results from the double-blind, randomized, placebo-controlled phases of the PREEMPT clinical program	USA, Germany	Dodick et al. (2010) [38]
7 (638)	22 (19)	Crystal structure of botulinum neuro-toxin type A and implications for toxicity	USA	Lacy et al. (1998) [39]
8 (608)	11 (23)	SV2 is the protein receptor for botulinum neurotoxin A	USA	Dong et al. (2006) [40]
9 (584)	64 (11)	Botulinum-A toxin for treating detrusor hyperreflexia in spinal cord injured patients: A new alternative to anticholinergic drugs? Preliminary results	Switzerland	Schurch et al. (2000) [41]
10 (531)	16 (21)	Early and selective loss of neuromuscular synapse subtypes with low sprouting competence in motoneuron diseases	Switzerland, France	Frey et al. (2000) [42]

*TC*_2024_: the total number of citations from Web of Science Core Collection since publication year to the end of 2024; *C*_2024_: number of citations of an article in 2024 only.

**Table 8:** Top 20 most frequently used words in highly cited article title and author keywords.

Words in Title	*TP*	*R* (%) n = 643	Author Keywords	*TP*	*R* (%) n = 296
Treatment	149	1 (23)	Spasticity	22	1 (6.6)
Double-blind	65	2 (10)	Cerebral palsy	18	2 (5.4)
Trial	52	3 (8.1)	Urinary incontinence	18	2 (5.4)
Patients	51	4 (7.9)	Bladder	16	4 (4.8)
Efficacy	42	5 (6.5)	Dystonia	15	5 (4.5)
Injection	42	5 (6.5)	Cervical dystonia	14	6 (4.2)
Chronic	40	7 (6.2)	Chronic migraine	13	7 (3.9)
Dystonia	38	8 (5.9)	Pain	12	8 (3.6)
Spasticity	38	8 (5.9)	Blepharospasm	11	9 (3.3)
Injections	35	10 (5.4)	Prophylaxis	11	9 (3.3)
Safety	34	11 (5.3)	Stroke	11	9 (3.3)
Pain	33	12 (5.1)	Botulism	10	12 (3.0)
Bladder	30	13 (4.7)	Inflammation	10	12 (3.0)
Migraine	30	13 (4.7)	Exocytosis	8	14 (2.4)
Clinical	27	15 (4.2)	Spinal cord injuries	8	14 (2.4)
Detrusor	27	15 (4.2)	Urinary bladder	8	14 (2.4)
Management	23	17 (3.6)	Achalasia	7	17 (2.1)
Cervical	22	18 (3.4)	Clostridium botulinum	7	17 (2.1)
Protein	22	18 (3.4)	Neurogenic bladder	7	17 (2.1)
Achalasia	21	20 (3.3)	Overactive	7	17 (2.1)

*TP*: number of articles; %: percentage; *R*: rank.

## Data Availability

No new data were created or analyzed in this study. Data sharing is not applicable to this article.

## Supplementary Materials

The following supporting information can be downloaded at: https://www.mdpi.com/article/10.3390/healthcare14111480/s1, Table S1: The BIBLIO checklist for reporting the bibliometric reviews of the biomedical literature [15].

## Abbreviations

The following abbreviations are used in this manuscript:

APPs	authors per publication
CPP	average number of citations per publication
FP	number of first-author articles
RP	number of corresponding-author articles
SCI-EXPANDED	Science Citation Index Expanded
TCs	total citations
TP	total number of publications
WoSCC	Web of Science Core Collection
